# The current status of foundation models in decoding inner speech from non-invasive brain signals: a mini review

**DOI:** 10.3389/fnhum.2026.1838064

**Published:** 2026-05-28

**Authors:** Esra Sümer-Arpak, Rajkumar Saini, Debashis Das Chakladar, Sanjeev Kumar Varun, Foteini Simistira Liwicki

**Affiliations:** Division of Embedded Intelligent Systems LAB, Department of Computer Science, Electrical and Space Engineering, Luleå University of Technology, Luleå, Sweden

**Keywords:** deep learning, foundation models, inner speech decoding, neural signals, non-invasive neuro imaging

## Abstract

Inner speech (IS), or imagined speech without overt articulation, is a promising target for brain-computer interfaces (BCIs) aimed at restoring communication in individuals with severe speech impairments, such as locked-in syndrome. Foundation models (FMs), typically trained using self-supervised learning (SSL) on large-scale datasets, offer new opportunities for learning transferable and robust representations from neural signals. This mini review provides an overview of FM-based approaches for IS decoding using non-invasive neuroimaging modalities, including functional magnetic resonance imaging, electroencephalography, magnetoencephalography, and functional near-infrared spectroscopy, highlighting architectural trends, pretraining strategies, and model adaptation techniques. We discuss how recent models move beyond task-specific classification toward scalable representation learning and semantic-level decoding. Despite these advances, several challenges remain, including the weak, noisy, and non-stationary nature of neural signals, variability in data acquisition, and limitations in dataset scale, standardization, computational resources, interpretability, and evaluation metrics. Ethical and privacy considerations are also critical. Overall, FMs provide a promising paradigm for non-invasive IS decoding, addressing neurophysiological, methodological, and ethical challenges is essential for developing scalable and reliable BCI systems.

## 1 Introduction

Speech is fundamental to daily communication, yet neurological disorders, trauma, and disease can impair this ability (Shah et al., 2022). Inner speech (IS), also referred to as verbal thinking or covert self-talk, describes the internal experience of language without overt or subvocal articulation (Alderson-Day and Fernyhough, 2015). IS encompasses related paradigms, including silent or intended speech (attempted articulation without sound), articulatory motor imagery (imagined speech), and phonological rehearsal (the internal repetition of speech sounds to support working memory) (Nieto et al., 2022; Schultz et al., 2017). These paradigms involve different neural systems and signal characteristics, affecting decoding performance. In this review, we adopt Vygotsky's model (Vygotsky, 1987), defining IS

as an internalized process of thinking in pure meanings, distinct from motor imagery or phonological rehearsal (Schultz et al., 2017). Decoding IS from non-invasive brain signals holds promise for brain–computer interfaces (BCIs) aimed at restoring communication in individuals with severe impairments, such as those with locked-in syndrome (LIS).

IS elicits activity in speech-related brain regions (Shergill et al., 2001). Its neural basis has been investigated using multiple neuroimaging modalities, including functional magnetic resonance imaging (fMRI), electroencephalography (EEG), magnetoencephalography (MEG), functional near-infrared spectroscopy (fNIRS), and electrocorticography (ECoG). Among them, ECoG provides high-resolution intracranial recordings of neural activity (Martin et al., 2018). Several studies have exploited these advantages to achieve higher decoding performance (Pei et al., 2011; Martin et al., 2016; Komeiji et al., 2024). However, it requires invasive electrodes, which limits its scalability and generalizability (Martin et al., 2018). In contrast, non-invasive modalities, despite typically yielding lower performance, offer scalable solutions and have demonstrated promising results for IS decoding (Almufareh et al., 2025). Detailed comparisons of these modalities, including their signal principles, spatial–temporal resolution, and associated trade-offs, are summarized in Supplementary Table S1. These differences affect decoding performance and motivate multimodal approaches and advanced analytical methods for reliable IS decoding (Cooney et al., 2022; Wellington et al., 2024).

Recent advancements in machine learning and computational power have strengthened links between cognitive neuroscience and practical applications, such as decoding IS from brain signals. IS decoding can be performed using traditional machine learning classifiers or deep learning models. Classical techniques such as support vector machines (SVM) (Wellington et al., 2024), Random forests (Hernández-Del-Toro et al., 2021), and gradient boosting machines (Pan et al., 2023) have been used for feature-based classification of IS. Conversely, deep learning models do not require manual feature extraction. Supervised deep learning models, such as convolutional neural networks (CNNs) (Simistira Liwicki et al., 2022), recurrent neural networks (RNNs) (Chengaiyan et al., 2020), and hybrid methods (Saha and Fels, 2019), have achieved remarkable results. However, supervised deep learning models need large labeled datasets, which limits the robustness and generalization of these models (Bommasani et al., 2021). Recently proposed foundational models (FMs) have gained significant attention because they are trained on pretext tasks with unlabeled data and can then be applied to multiple downstream tasks (Gui et al., 2024). These approaches have already proven successful in learning robust representations from brain signals and are increasingly explored for IS decoding (Lesaja et al., 2022).

This mini review summarizes recent representative studies employing FM-based approaches using non-invasive neuroimaging modalities and their potential applicability to IS decoding. Relevant studies were identified through major databases (e.g., IEEE Xplore, PubMed, Scopus, and Google Scholar), focusing on publications from 2022 to 2025. As self-supervised learning (SSL) underpins most FMs, SSL-based non-FM approaches were also included. We highlight key developments, challenges, and future directions for advancing robust and transferable IS decoding systems.

## 2 Framework of foundation models for neural signal processing

**Figure 1:**
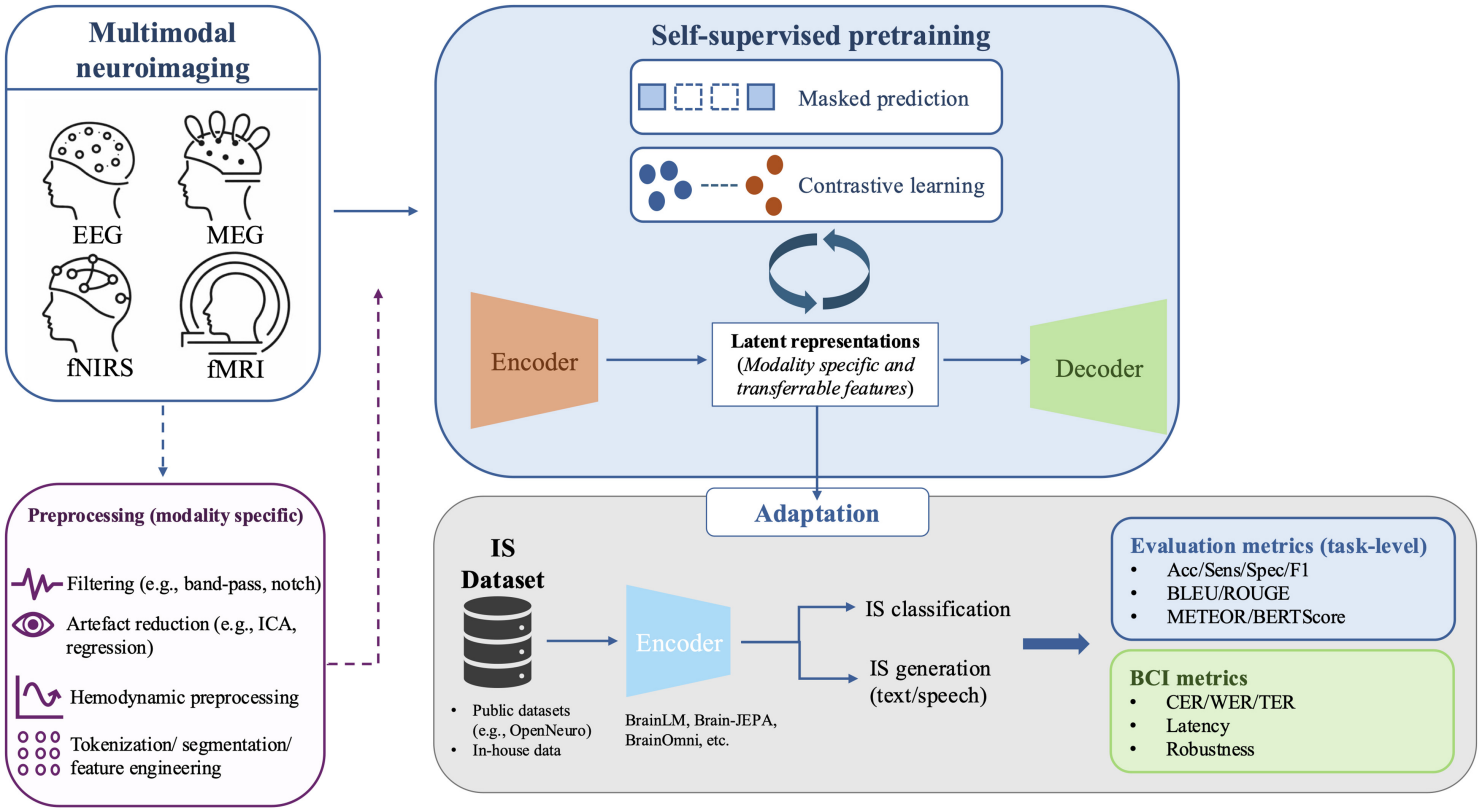
Multimodal FM-based framework for IS decoding, illustrating modality-specific preprocessing, self-supervised pretraining, representation learning, and adaptation to IS tasks. The figure highlights differences between task-level evaluation metrics and application-level BCI performance measures along with representative FM-based approaches.

FMs have emerged as a major paradigm in artificial intelligence (AI), enabling learning from large-scale unlabeled data and adaptation to multiple downstream tasks (Bommasani et al., 2021). Figure 1 illustrates the overall framework of FMs for neural signal processing, including preprocessing (if applied), self-supervised pretraining, downstream adaptation, and task-dependent evaluation metrics.

- Input data: The models are trained on broad and often unstructured datasets to capture a general representation. More specifically, brain-signal datasets are high-dimensional, temporally rich, exhibit high noise, and vary across subjects and data acquisition conditions (Zhou et al., 2025b). Although FMs can reduce reliance on expensive manual annotation, neural recordings (e.g., EEG and fMRI) are highly irregular and dynamic, making it difficult to impose a consistent structure for pretraining (Craik et al., 2019; Wang et al., 2025a).
- Self-supervised learning: FMs build upon a training strategy called SSL, which leverages intrinsic information in the data by creating pretext tasks, either generative or contrastive, such as predicting masked inputs or reconstructing corrupted signals to make the model learn informative latent representations. FMs also adopt another subclass of SSL training strategy, contrastive learning, which aims to learn discriminative representations of data by using negative and positive pairs (Chen et al., 2020). FMs are trained on broad datasets (large language models (LLMs), videos, images, brain signals, structured data) with SSL or unsupervised techniques to be adaptable to downstream tasks (Bommasani et al., 2021).
- Architecture: Designing network architecture is another key factor that influences FM's ability to capture and encode relevant information from the raw signal (Bommasani et al., 2021). The neural-signal FMs aim to (i) capture local signal patterns, (ii) model cross-channel interactions while remaining robust to noise and artifacts, and (iii) scale to temporal contexts to learn broader dependencies.
  Neural-signal FMs usually employ tokenization layers, local representation learning block, spatiotemporal attention mechanisms, and downstream task heads (Kuruppu et al., 2025). To handle the non-stationary nature of neural signals, FMs can adopt a signal segmentation method to transform continuous time series into structured representations, a process referred to as tokenization. Depending on model architecture and signal, tokenization strategies (window length, overlap vs. non-overlap patches, etc.) can be optimized for increasing computational efficiency and better modeling performance (Gu et al., 2025). On the other hand, tokenization provides a unified representation of raw signals and placed into a common input format, which can obscure or weaken the spatiotemporal information. To mitigate this, positional embeddings are utilized and added to the projected input to preserve temporal and spatial structure (Zhou et al., 2025b).
  Neural-signal FMs are typically built using several non-linear transformer blocks that can capture both local patterns and long-range dependencies. Moreover, transformer models provide more effective model parallelism, which makes them well-suited to large-scale datasets. However, non-transformer models such as CNN or CNN-transformer hybrids can be good choices for avoiding potential overfitting through exploiting the spatial inductive bias of convolutions (Wu et al., 2021). Additionally, neural-signal FMs may adopt different configurations: encoder-only models are conceptually described as comprising two high-level functional components: a backbone encoder and a final fully connected layer. The backbone acts as a feature extractor, transforming the inputs into lower-dimension representations. These embeddings are then passed to a task head, typically a single fully connected layer, which produces task-specific outputs such as classification or regression (Kuruppu et al., 2025). In contrast, encoder-decoder hybrid models include an additional component: a decoder module that generates outputs conditioned on encoder representations, usually well-suited for generative or reconstruction-based tasks (e.g., neural signal-to-text or neural signals-to-speech embeddings) (He et al., 2022; Lewis et al., 2020).
- Model adaptation: The pretrained backbone enables reuse and transfer of neural-signal representations across multiple downstream tasks with related semantic structure. The degree of adaptation can be adjusted depending on data availability and domain shift; for instance, the backbone may be kept fully frozen and used only as a feature extractor or updated using parameter-efficient fine-tuning (PEFT) approaches (Houlsby et al., 2019) that update only a small subset of parameters (Kottapalli et al., 2025).

## 3 Foundation models in non-invasive neuroimaging and inner speech decoding

Recent works have increasingly applied FM principles to non-invasive neuroimaging modalities, including EEG, MEG, fMRI, and fNIRS. In this review, we adopt an operational definition of FMs based on large-scale pretraining, SSL paradigm, and transferability across downstream tasks, categorizing models as FMs or SSL/non-FM (Table 1). While only a subset directly targets IS decoding, others were included for their transferable representations. Additionally, IS studies vary substantially in task complexity, ranging from closed-set classification to continuous open-vocabulary generation, which is substantially more challenging for non-invasive IS decoding.

**Table 1:** Summary of representative IS decoding and SSL/FM-based approaches across neuroimaging modalities.

Study	Modality	Task type	Task/application	Performance	Notes
SSL/non-FM					
Shuzo et al. (2025)	EEG	Classification	IS decoding (Tasin et al., 2024)	Acc = 16.5%; F1 = 0.137	Transformer (BERT based); closed-set (6-class IS); cross-subject; low performance
Wang et al. (2024)	EEG	Generation	EEG-to-text decoding (Hollenstein et al., 2018, 2023)	BLEU-1: 42.09%; BLEU-4: 8.99%; ROUGE-1 F1: 32.61%	Multi-stream transformer; open vocabulary (reading-based EEG-to-text, not specific to IS)
Zhou et al. (2025a)	EEG	Representation learning and classification	IS decoding	Semantic acc: 47.0%, word acc: 39.6%	FM-like characteristics; FSTP pre-training; closed-set vocabulary
Lee et al. (2023)	EEG	Generation	IS decoding	CER = 68.26%; MOS = 2.78; RMSE = 0.175	Spoken EEG supervision (domain adaptation); supports unseen word generation (limited performance); closed-set (12-class IS)
Jayalath et al. (2024)	MEG	Decoding	Perceived speech (Armeni et al., 2022; Gwilliams et al., 2023)	ROC AUC = 0.705	SSL-based transformer; perceived speech classification (closed-set); near surgical-level performance
Millet et al. (2022)	fMRI	Representation learning and decoding	Speech perception (Nastase et al., 2019; Li et al., 2021)	Correlation (R) ~0.20	SSL (wav2vec 2.0); not IS-specific; listening task
FM					
Jiang et al. (2024)	EEG	Representation learning and classification	Abnormality detection and event classification (Obeid and Picone, 2016)	Task-dependent; improves downstream performance	Large scale pre-trained EEG FM
Caro et al. (2024)	fMRI	Representation learning and classification	Clinical variable and brain state prediction (Miller et al., 2016; Elam et al., 2021)	Task-dependent; improves performance	Not IS-specific
Dong et al. (2024)	fMRI	Multitask and representation learning	Clinical variable and neurodegenerative disease prediction (Miller et al., 2016; Jack Jr et al., 2008)	Task-dependent; improves performance	Not IS specific; transferable
Wang et al. (2025b)	fMRI	Multitask and representation learning	Clinical classification (HD-200 Consortium, 2012; Jack Jr et al., 2008; Marek et al., 2011; Di Martino et al., 2014) and Asian participants (MACC)	Task-dependent; improves performance	Not IS-specific
Wang et al. (2025a)	fMRI	Multitask and representation learning	Phenotype and diagnosis prediction (Van Essen et al., 2013; Meling et al., 2024)	Task-dependent; improves performance	Not IS-specific; transferrable
Wei et al. (2025)	fMRI	Representation learning and generation	Phenotype and fMRI-to-text (Elam et al., 2021; Miller et al., 2016; Karcher and Barch, 2021)	Task-dependent; strong generalization	Not IS specific; language-aligned; potential relevance to IS
Jung et al. (2025)	fNIRS	Representation learning and classification	Cognitive classification	Task-dependent; acc ~0.67–0.79	Data-efficient; not IS specific; transferrable
Zhang S. et al. (2024)	fNIRS	Generation	IS decoding (4 participants)	BLEU-1 = 0.25; BERTScore = 0.88; METEOR = 0.17; ROUGE-L = 0.21; WER = 0.84 (mean across subjects)	Small sample size; prompt-tuned LLM integration; continuous and open-vocabulary; limited lexical acc and moderate semantic decoding
Ferrante et al. (2025)	EEG, MEG, and fMRI	Representation learning and decoding	Decoding, encoding, modality conversion	Task-dependent; improves cross-modal performance	Not IS specific; transferable across modalities
Xiao et al. (2025)	EEG and MEG	Representation learning and classification	Multiple datasets (clinical, motor, multimodal)	Balanced acc; outperforms baselines	Not IS specific; large-scale multimodal FM

The table distinguishes between SSL/non-FM and FM approaches, highlighting differences in task types, applications, datasets, and performance metrics.

Acc, Accuracy; AUC-PR, area under the precision–recall curve; ROC AUC, receiver operating characteristic area under the curve; RMSE, root mean squared error; CER, character error rate; MOS, mean opinion score; MACC, Memory, Ageing and Cognition Centre; LLM, large language model; WER, word error rate; FSTP, future spectro-temporal prediction; BLEU, Bilingual Evaluation Understudy; BERTScore, Bidirectional Encoder Representations from Transformers Score; METEOR, Metric for Evaluation of Translation with Explicit Ordering; ROUGE, Recall-Oriented Understudy for Gisting Evaluation.

In electrophysiological modalities such as EEG and MEG, large-scale pre-trained models including Large Brain Model (LaBraM) (Jiang et al., 2024) and Large Brain Language Model (LBLM) (Zhou et al., 2025a) have aimed to learn generic and transferable representations from heterogeneous multi-subject datasets. These models are adapted to tasks such as classification and IS decoding (Zhou et al., 2025a). Building on this direction, transformer-based architectures have also been explored for EEG-based IS decoding. For example, (Shuzo et al. 2025) applied a pre-trained lightweight BERT model (Devlin et al., 2019) for six-class IS classification; however, the reported performance (accuracy = 0.165, F1 = 0.137) is close to chance level, indicating limited performance in EEG-based IS decoding. Other approaches, such as contrastive EEG–text masked autoencoders (CET-MAE) (Wang et al., 2024), have introduced language-aligned representation learning, using EEG embeddings to map into shared semantic spaces with textual representations. Recent pre-training paradigms, including Future Spectro-Temporal Prediction (Zhou et al., 2025a), and generative frameworks such as NeuroTalk (Lee et al., 2023), have further explored potentially transferable representation learning for IS, primarily in limited-vocabulary settings. A complementary MEG study has also suggested that, rather than using handcrafted features and small supervised models, scaling data and SSL may improve cross-subject speech decoding performance (Jayalath et al., 2024). Although the method was demonstrated primarily for decoding perceived speech rather than IS, the study can contribute to the critical step of cross-subject decoding of IS technologies.

In fMRI research, similar trends toward representation learning have been proposed. Models such as BrainLM (Caro et al., 2024), Brain-JEPA (Dong et al., 2024), SLIM-Brain (Wang et al., 2025b), and NeuroSTORM (Wang et al., 2025a) have marked a shift from task-specific prediction toward learning general cortical representations. In this direction, fMRI-LM (Wei et al., 2025) has incorporated large-scale, multi-subject pretraining aligned with pretrained language models, aiming to improve transferability across subjects. Converging evidence from speech models, Millet et al. demonstrated that SSL speech models, such as wav2vec 2.0 (Baevski et al., 2020), exhibited hierarchical representations that align with distributed cortical speech processing patterns (Millet et al., 2022). These findings suggested that the proposed models capture biologically meaningful and hierarchically structured speech representations.

Emerging FM approaches have been explored in hemodynamic neuroimaging beyond fMRI. For example, fNIRS-based models (Jung et al., 2025) have demonstrated that scalable pre-training strategies can extend to hemodynamic modalities. Recent systems, such as MindSpeech (Zhang S. et al., 2024), have employed pretrained language models to enable continuous, open-vocabulary, and semantically informed decoding. However, the reported performance indicates low lexical accuracy despite moderate semantic similarity, suggesting that current performance remains below clinically viable thresholds for reliable communication. These limitations highlight the constraints of single-modality decoding and motivate a shift toward unified frameworks that integrate complementary non-invasive neural modalities (Ferrante et al., 2025). For instance, BrainOmni has been designed to generalize across EEG and MEG recordings, learning shared spatio-temporal representations (Xiao et al., 2025). The model has aimed to capture modality-invariant neural dynamics across EEG and MEG. This cross-modality framework may enable knowledge transfer for IS decoding.

## 4 Discussion

Despite rapid advances in FM approaches for BCI, several structural, methodological, and practical obstacles persist, limiting scalable and reliable IS decoding.

### 4.1 Scale, computational constraints, and data heterogeneity

FMs benefit from massive, diverse datasets to learn latent structural patterns in neural data through self-supervised objectives. However, in neuroimaging research, data collection remains limited and costly (Wang et al., 2025c). In addition, training high-capacity spatiotemporal models on neuroimaging data is computationally demanding.

Beyond scale, neural data are inherently heterogeneous and non-stationary. Variability in acquisition protocols, scanner parameters, preprocessing pipelines, and experimental paradigms complicates data aggregation and large-scale pretraining. Neural recordings are also vulnerable to artifacts, further challenging robustness. These issues become more pronounced in multimodal settings, where EEG, MEG, fMRI, and fNIRS require harmonizing signals with fundamentally different spatial and temporal properties (Zhou et al., 2025b).

Recent large-scale datasets, such as LibriBrain, comprising over 50 h of MEG recordings during speech processing (Özdogan et al., 2025) and MOUS, which includes multimodal recordings from 204 subjects (Schoffelen et al., 2019), represent important steps toward scalable neural speech modeling. However, datasets specifically designed for IS are typically limited to single-modality recordings and relatively small cohorts (Nieto et al., 2022) with only a few studies acquiring simultaneous multimodal neural data (Cooney et al., 2022; Liwicki et al., 2025). Although recent efforts such as the Chisco dataset have introduced larger-scale EEG-based imagined speech corpora comprising semantically diverse daily expressions (Zhang Z. et al., 2024), IS datasets remain limited in subject diversity and recording modalities.

Moreover, IS datasets remain limited in language diversity (Simistira Liwicki et al., 2023; Zhang Z. et al., 2024). This limitation is particularly important, as language shapes the neural representation of IS. For instance, linguistic properties such as morphological complexity, tonal structure, and word segmentation may produce distinct cortical activation patterns (Dash et al., 2020). Efforts to extend IS decoding to multiple languages underscore the importance of linguistic diversity in datasets and the systematic analysis of cross-linguistic differences in neural signals to identify universal neural correlates of covert speech (Almufareh et al., 2025). Addressing these challenges will require standardized data sharing (e.g., OpenNeuro), harmonized preprocessing pipelines, standardized data formats such as Brain Imaging Data Structure (BIDS) (Gorgolewski et al., 2016), consistent experimental paradigms, and scalable training strategies tailored to neuroimaging data.

Overcoming these limitations is essential for enabling effective large-scale FM, facilitating cross-subject and cross-lingual generalization, and ultimately learning robust, invariant, and generalizable neural representations for IS decoding.

### 4.2 Neurophysiological constraints of IS and implications for FMs

Decoding the content of IS, i.e., internally generated speech from complex neural activity, presents several task-specific challenges. First, neural signals associated with IS are typically weaker and noisier than those observed in overt or attempted speech (Almufareh et al., 2025). Although IS has been associated with internal motor predictions such as efference copies (Whitford et al., 2017), the absence of overt articulation limits the availability of external timing markers and the predictability of neural signals (Wang et al., 2025c).

Furthermore, non-invasive IS decoding is particularly constrained by low signal-to-noise ratios (e.g., in scalp EEG), the distributed and complex cortical encoding of speech, and substantial inter-subject variability. From an FM perspective, these challenges highlight the need for SSL approaches capable of learning invariant spatiotemporal representations from weak, noisy, and non-stationary neural signals. In addition, developing robust decoders that can disentangle variability in neural representations arising from neurological conditions (e.g., stroke and LIS), as well as differences in cognitive profiles, remains a central challenge in the field.

### 4.3 Evaluation metrics and interpretability

Traditional performance metrics, such as classification accuracy and F1 score, are suitable for closed-set classification tasks but are not sufficient to capture semantic fidelity in sentence-level decoding. In generative frameworks, metrics such as Bilingual Evaluation Understudy (BLEU) (Papineni et al., 2001) and Recall-Oriented Understudy for Gisting Evaluation (ROUGE) (Lin, 2004) provide assessments of exact textual overlap. However, these metrics do not fully capture semantic fidelity. For more semantically informed evaluation, the Metric for Evaluation of Translation with Explicit Ordering (METEOR) (Banerjee and Lavie, 2005) and BERTscores (Zhang et al., 2019) have been employed. Furthermore, practical BCI systems are typically assessed by performance measures such as bit rate, character error rate (CER), word error rate (WER), average token error rate (TER), and latency, which reflect communication efficiency and real-time usability (He et al., 2026). Nevertheless, the lack of standardized evaluation protocols that reliably reflect deeper conceptual meaning and provide a concise summary of model performance hinders fair comparisons in IS decoding research.

Another critical bottleneck lies in the interpretability of FM-based decoders. Deep learning models capture complex non-linear patterns that are difficult to interpret in neural terms and therefore often work as “black-box” systems. Combined with cortical complexity and inter-subject variability, this limits explainability and raises concerns about trust and ethical deployment (Doshi-Velez and Kim, 2017). Methods such as attention and saliency visualizations offer some degree of insight about models' decisions; however, they often do not provide enough detail to understand the mechanism behind a model's decisions and lack of neuroscientific ground (Rudin, 2019). More neuroscientific approaches, such as Representational Similarity Analysis (RSA), can compare the similarity structure of the model embeddings with cortical activity patterns (Kriegeskorte, 2008). Developing inherently interpretable frameworks that provide transparent outputs and clear feedback while aligning with model-derived neural representation with neuroscience principles remains an important challenge.

### 4.4 Continuous and real-time IS decoding

Real-world BCI applications require FM-based decoders capable of processing weak, non-stationary neural signals in real time. However, the absence of overt behavioral anchors in IS complicates onset detection and poses a fundamental challenge for continuous, open-vocabulary decoding (Martin et al., 2014). Moreover, non-invasive modalities provide indirect and spatially coarse measurements of neural activity, limiting their ability to capture high-frequency articulatory representations associated with speech production. Designing FMs that balance computational efficiency, robustness, and adaptability, therefore, remains an open challenge for scalable BCI systems.

In contrast, invasive BCI systems based on ECoG have demonstrated continuous speech decoding (Anumanchipalli et al., 2019) and, in some cases, near real-time operation (Metzger et al., 2023; Moses et al., 2021), achieving substantially higher performance and larger vocabularies. These systems leverage high-frequency cortical activity directly linked to speech production, resulting in significantly higher signal fidelity. Although most results have been derived from overt or attempted speech rather than IS, they have established a practical upper bound for decoding performance. This highlights a gap between invasive and non-invasive approaches, reflecting differences in signal accessibility and posing a key limitation for achieving continuous IS decoding with non-invasive FMs.

### 4.5 Ethical and privacy considerations

Ethical and privacy considerations are central to the development of IS decoding technologies. Neural data are highly sensitive and subject to strict privacy and institutional constraints. Unlike many other biomedical signals, brain-derived data may reveal not only speech content but also aspects of a participant's mental state (Ienca and Andorno, 2017). This concern is particularly important for IS decoding, as IS is widely described as an intrinsically private mental process through which individuals plan, reflect, and encode representations that guide behavior (Alderson-Day and Fernyhough, 2015). Accordingly, strong privacy safeguards and transparent frameworks are essential. Emerging technical solutions, such as federated learning, can enable collaborative model training without centralized data sharing, while biologically informed synthetic data augmentation may reduce reliance on raw neural recordings (Kwon and Shin, 2026). Transparency and explainability are also critical for strengthening public trust in IS technologies and addressing concerns related to “mind reading.” Robust frameworks for informed consent, user agency, and clearly defined privacy boundaries remain essential prerequisites for responsible deployment (Wang et al., 2025c).

As these technologies transition to real-world applications, broader ethical and societal considerations become increasingly important. Issues of access, regulation, and responsible use must be carefully addressed, particularly regarding potential biases, unequal access, and the amplification of existing social inequalities. Addressing these challenges will require interdisciplinary collaboration among engineers, ethicists, clinicians, and policymakers to ensure that technological advances are aligned with appropriate governance frameworks and responsible deployment (Almufareh et al., 2025).

In conclusion, FMs are increasingly applied to non-invasive neuroimaging for IS decoding. Through large-scale self-supervised pretraining and transferable representations, they offer a promising alternative to data-intensive supervised approaches. These models may improve cross-subject generalization, capture contextual dependencies, and mitigate data scarcity. However, key challenges remain, including limited dataset scale, neurophysiological constraints, inconsistent evaluation metrics (especially for semantic decoding), interpretability, and ethical concerns. Achieving continuous, real-time IS decoding with robust, transparent, and privacy-preserving systems remains difficult. Bridging advances in FMs, neuroscience, and human–computer interaction will be essential for developing scalable and trustworthy IS decoding systems.
