# Supplementary material for: The current status of foundation models in decoding inner speech from non-invasive brain signals: a mini review
Source: Front Hum Neurosci. 2026 May 28;20:1838064. doi: 10.3389/fnhum.2026.1838064 (PMC13254259; doi:10.3389/fnhum.2026.1838064)
Supplement: Supplementary file 1 [file Data_Sheet_1.pdf]

Supplementary Material

1 SUPPLEMENTARY DATA

Table S1. Comparison of neuroimaging modalities.

Modality	Principle	Spatial Resolution	Temporal Resolution	Major Strengths	Major Limitations
EEG	Electrical potentials from neural currents	Low (cm)	High (ms)	High temporal resolution, relatively low cost, portable	Low spatial specificity, high susceptibility to artefacts/noise
MEG	Magnetic fields from neural currents	Moderate (mm–cm)	High (ms)	High temporal resolution with improved spatial localization compared to EEG	Expensive, low portability, requires shielded room
fMRI	Hemodynamic (BOLD) signal	High (mm)	Low (seconds)	High spatial resolution	Poor temporal resolution, expensive, immobile
fNIRS	Hemodynamic (oxygen changes)	Low–moderate (cm)	Moderate (seconds)	Balance of resolution, portable	Lower spatial resolution than fMRI, slower than MEG/EEG
